# Low-affinity TCR engagement drives IL-2-dependent post-thymic maintenance of naive CD4+ T cells in aged humans

**DOI:** 10.1111/acel.12353

**Published:** 2015-05-25

**Authors:** Kornelis S M van der Geest, Wayel H Abdulahad, Nato Teteloshvili, Sarah M Tete, Jorieke H Peters, Gerda Horst, Pedro G Lorencetti, Nicolaas A Bos, Annechien Lambeck, Caroline Roozendaal, Bart-Jan Kroesen, Hans J P M Koenen, Irma Joosten, Elisabeth Brouwer, Annemieke M H Boots

**Affiliations:** 1 Department of Rheumatology and Clinical Immunology, University of Groningen, University Medical Center GroningenHanzeplein 1, 9713 GZ, Groningen, The Netherlands; 2 Department of Pathology and Medical Biology, University of Groningen, University Medical Center GroningenHanzeplein 1, 9713 GZ, Groningen, The Netherlands; 3 Department of Laboratory Medicine – Medical Immunology, Radboud University Medical CentrePostbus 9101, 6500 HB, Nijmegen, The Netherlands; 4 Department of Laboratory Medicine, University of Groningen, University Medical Center GroningenHanzeplein 1, 9713 GZ, Groningen, The Netherlands

**Keywords:** aging, homeostasis, interleukin-2, interleukin-7, TCR engagement, T lymphocytes

## Abstract

Insight into the maintenance of naive T cells is essential to understand defective immune responses in the context of aging and other immune compromised states. In humans, naive CD4+ T cells, in contrast to CD8+ T cells, are remarkably well retained with aging. Here, we show that low-affinity TCR engagement is the main driving force behind the emergence and accumulation of naive-like CD4+ T cells with enhanced sensitivity to IL-2 in aged humans. *In vitro,* we show that these CD45RA^+^CD25^dim^CD4^+^ T cells can develop from conventional naive CD25^−^CD4+ T cells upon CD3 cross-linking alone, in the absence of costimulation, rather than via stimulation by the homeostatic cytokines IL-2, IL-7, or IL-15. *In vivo*, TCR engagement likely occurs in secondary lymphoid organs as these cells were detected in lymph nodes and spleen where they showed signs of recent activation. CD45RA^+^CD25^dim^CD4+ T cells expressed a broad TCRVβ repertoire and could readily differentiate into functional T helper cells. Strikingly, no expansion of CD45RA^+^CD25^dim^CD8+ T cells was detected with aging, thereby implying that maintenance of naive CD4+ T cells is uniquely regulated. Our data provide novel insight into the homeostasis of naive T cells and may guide the development of therapies to preserve or restore immunity in the elderly.

## Introduction

A broad naive T cell repertoire is essential for optimal immunity against novel antigenic challenges (Nikolich-Zugich *et al*., 2004; Goronzy *et al*., 2007; Blackman & Woodland, 2011). Contractions of the T cell repertoire have been linked to poor immunity in the context of aging, stem cell transplantation, cancer, and HIV infection (Gorochov *et al*., 1998; Maury *et al*., 2001; Saurwein-Teissl *et al*., 2002; Baum *et al*., 2012; Manuel *et al*., 2012). To develop strategies for preserving and restoring the naive T cell repertoire, insight into the basic mechanisms driving naive T cell homeostasis is critical.

So far, most knowledge on the homeostasis of naive T cells is derived from mouse studies (Surh & Sprent, 2008). Mechanisms of naive Tcell maintenance, however, differ substantially in mice and men (den Braber *et al*., 2012). Whereas thymic output in mice is sustained on to high age, thymic involution drastically reduces T cell replenishment in adult humans (den Braber *et al*., 2012). Maintenance of the naive T cell pool in humans therefore relies on low-grade proliferation and long-term survival of already existing naive T cells in the periphery (Bains *et al*., 2009; den Braber *et al*., 2012). Furthermore, a clear difference has been noted between the maintenance of naive CD4+ and CD8+ T cells in humans. (Goronzy *et al*., 2007). Whereas circulating numbers of naive CD8+ T cells decline with age, naive CD4+ T cells are remarkably well retained (Wertheimer *et al*., 2014). So far, little is known about the mechanisms underlying this difference.

Two types of signals are thought to drive the peripheral homeostasis of naive CD4+ T cells in humans (Kohler & Thiel, 2009). The first are T-cell receptor (TCR)-derived signals, as evidenced by an increase in naive CD4+ T cells lacking CD31 in aged humans (Kimmig *et al*., 2002). CD31 (PECAM-1) is expressed by recent thymic emigrant naive CD4+ T cells (CD31+ naive CD4+ T cells) but is lost upon TCR-derived signaling only (Kohler *et al*., 2005; Azevedo *et al*., 2009). In addition, homeostatic cytokines can promote the maintenance of naive CD4+ T cells. IL-7 promotes the expansion and survival of human, naive CD4+ T cells (Sportes *et al*., 2008). Other cytokines such as IL-2 and IL-15 have long been thought less important for the homeostasis of naive CD4+ T cells (Rochman *et al*., 2009).

Recently, a novel population of naive CD4+ T cells expressing the IL-2Rα chain (CD25) was observed in peripheral blood of aged humans (Pekalski *et al*., 2013). These naive CD4+ T cells were characterized by an enhanced response to IL-2 and were thought to develop after IL-7 stimulation (Pekalski *et al*., 2013). Yet, important questions regarding the origin and functional relevance of these cells remain to be answered. Here, we performed an extensive phenotypical and functional analysis of aging-associated CD25-expressing naive CD4+ T cells. Furthermore, the effects of TCR stimulation and homeostatic cytokines on the *in vitro* development of CD25-expressing naive CD4+ T cells were evaluated. To elucidate where CD25-expressing naive CD4+ T cells may develop *in vivo*, different types of human, lymphoid tissues were analyzed. Finally, we assessed the functionality of CD25-expressing naive CD4+ T cells in aged humans.

## Results

### Maintenance of naive CD4+ T cells but not naive CD8+ T cells in aged humans

**Fig 1:**
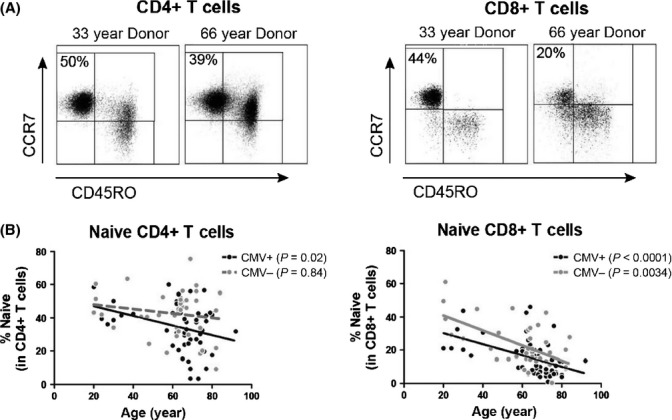
Maintenance of naive CD4+ T cells and loss of naive CD8+ T cells in aged humans. (A) Representative flow cytometric staining of naive (CD45RO-CCR7+) CD4+ and CD8+ T cells in a young and aged individual. The percentage of naive cells among CD4+ and CD8+ T cells is shown. (B) Proportions of naive CD4+ and CD8+ T cells as a function of age in 52 CMV-seropositive and 39 CMV-seronegative healthy, adult humans. Donor ages ranged between 20 and 92. Correlations were tested with Spearman’s rank correlation coefficient. *P* values are shown in the graph.

We first assessed the numbers of naive CD4+ and CD8+ T cells in peripheral blood of healthy adults of different ages. Donors were divided according to CMV serostatus, as CMV infection may influence naive T-cell numbers (Wertheimer *et al*., 2014). Naive CD4+ and CD8+ T cells were identified as CD45RO-CCR7+ cells (Fig.1A), co-expressing CD45RA and CD27 (data not shown). As recently reported (Wertheimer *et al*., 2014), the proportions and absolute numbers of naive CD8+ T cells correlated inversely with age in both CMV-seropositive and CMV-seronegative individuals (Figs1B and S1). In contrast, the proportions and absolute numbers of naive CD4+ T cells only demonstrated an aging-associated decrease in CMV-seropositive, but not in CMV-seronegative, individuals. Thus, aging by itself is associated with a loss of naive CD8+ T cells, but not naive CD4+ T cells.

### CD45RA+CD25^dim^CD4+ T cells accumulate in the circulation of aged humans

**Fig 2:**
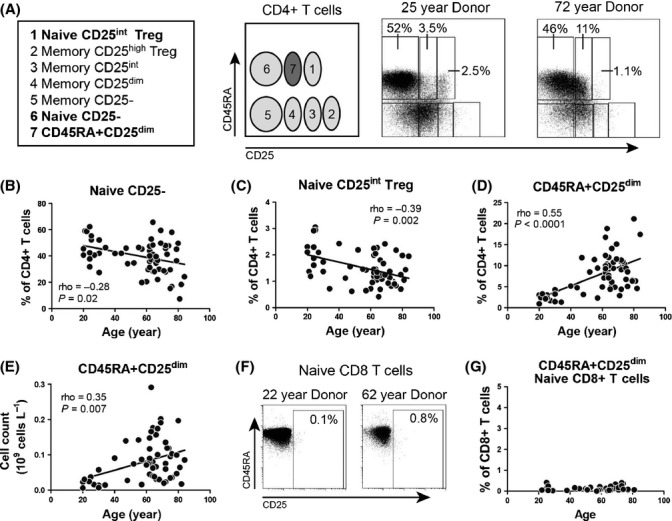
Accumulation of CD45RA+CD25^dim^CD4+ T cells in peripheral blood of aged humans. (A) Flow cytometric gating strategy for analysis of CD45RA and CD25 defined subsets in peripheral blood, as reported by Miyara *et al*. (Miyara *et al*., 2009). Representative flow cytometry plots are shown for a young and an aged individual. (B) Proportions of circulating naive CD25-CD4 T cells, (C) naive CD25^int^ regulatory T (Treg) cells, and (D) CD45RA+CD25^dim^CD4+ T cells in 63 healthy, adult humans of different ages. (E) Absolute numbers of CD45RA+CD25^dim^CD4+ T cells in 58 individuals of different ages. (F) Representative staining for CD45RA and CD25 in naive CD45RO-CD27+CD8+ T cells of a young and aged individual. (G) Proportions of circulating naive CD45RA+CD25^dim^CD8+ T cells in 44 healthy, adult humans of different ages. Correlations were assessed with Spearman’s rank correlation coefficient.

Next, we sought to confirm that aging is associated with an increase in CD25-expressing naive CD4+ T cells (Pekalski *et al*., 2013). Therefore, a CD45RA/CD25-based gating strategy allowing delineation of functionally distinct populations of naive and memory CD4+ T cells was applied (Miyara *et al*., 2009). Naive CD25-CD4+ T cells and naive CD25^int^ regulatory T (Treg) cells were readily detected in peripheral blood of adult humans (Fig.2A) (Miyara *et al*., 2009). The proportions of both cell populations, however, gradually decreased with age (Fig.2B and C). Interestingly, the aging-associated increase in CD25-expressing naive CD4+ T cells could be entirely attributed to the development of CD45RA+CD25^dim^CD4+ T cells. Not only the proportions but also the absolute numbers of CD45RA+CD25^dim^CD4+ T cells increased with age (Fig.2D and E). CD45RA+CD25^dim^CD4+ T cells accumulated in aged humans irrespective of gender and CMV serostatus (Fig. S2). Although some dim expression of CD25 was observed among CD45RA+CD8+ T cells (Fig.2F), CD45RA+CD25^dim^CD8+ T cells did not accumulate in the peripheral blood of aged humans (Fig.2G). Thus, the expansion of CD45RA+CD25^dim^cells with aging was restricted to the CD4+ T cell compartment.

### CD45RA+CD25^dim^CD4+ T cells display a naive phenotype

Additional phenotypical analysis showed that CD45RA+CD25^dim^CD4+ T cells were not late-stage memory T cells that re-express CD45RA and lack CD27 and CD28 (Fig. S3A). Like naive CD25- cells, CD45RA+CD25^dim^CD4+ T cells showed high expression of CCR7 and limited expression of CXCR3 and CCR6 (Fig. S3B). These findings indicated that CD45RA+CD25^dim^CD4+ T cells are indeed naive CD4+ T cells. Furthermore, CD45RA+CD25^dim^CD4+ T cells could produce some IL-2, but not IFN-γ, IL-4, or IL-17, upon short-term stimulation with PMA and calcium ionophore (Fig. S3C). This cytokine profile was similar to that of naive CD25-CD4+ T cells. CD45RA+CD25^dim^CD4+ T cells also lacked expression of Treg cell markers FOXP3, Helios, and IL-10, as well as the activation marker CD69 (Fig. S3D and E). Thus, CD45RA+CD25^dim^CD4+ T cells represented naive CD4+ T cells rather than Treg cells or recently activated T cells.

### CD45RA+CD25^dim^CD4+ T cells show signs of prior TCR engagement

**Fig 3:**
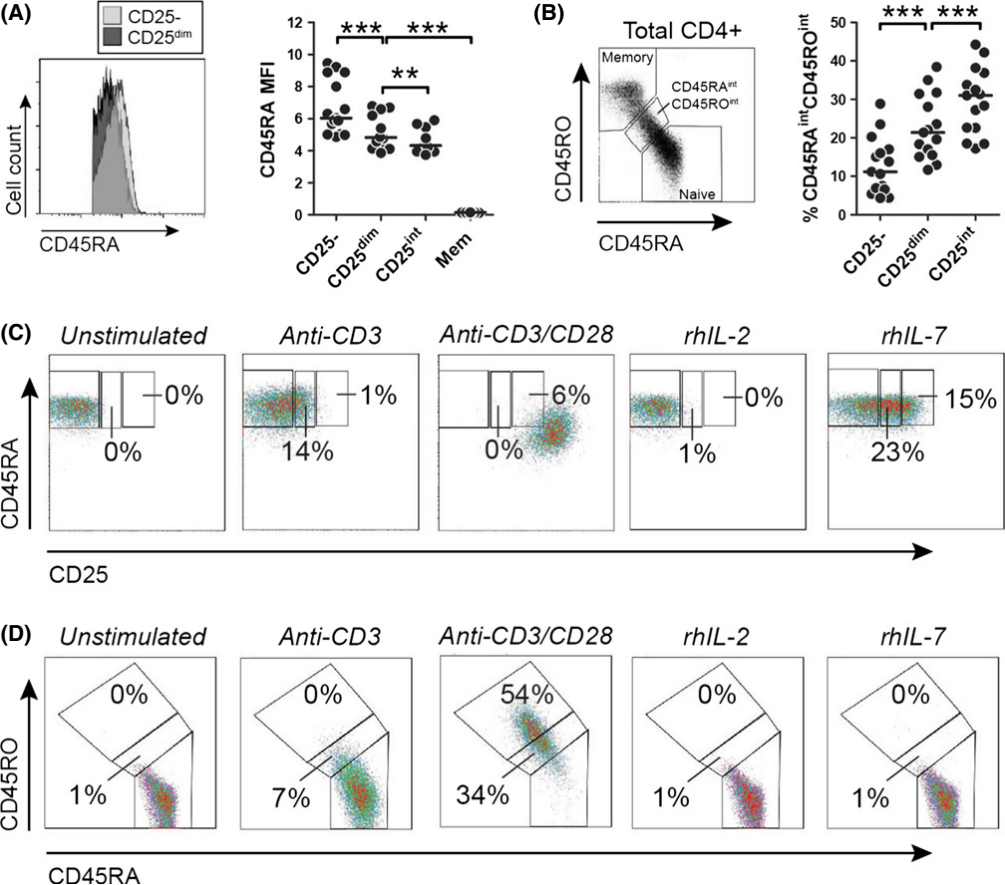
CD45RA+CD25^dim^CD4+ T cells show signs of prior TCR engagement. (A) Flow cytometric staining for CD45RA in CD45RA+CD25^dim^ and naive CD25- CD4+ T cells (left panel) and mean fluorescence intensity (MFI) of CD45RA in naive CD25-CD4+ T cells, CD45RA+CD25^dim^ CD4+ T cells, naive CD25^int^ regulatory T cells, and memory (Mem) CD4+ T cells of 15 aged individuals. (B) Gating for CD45RA^int^ CD45RO^int^ CD4+ T cells (left panel) and proportions of these cells in the 3 CD45RA+CD4+ T-cell subsets of aged individuals. (C) Development of CD45RA+CD25^dim^ cells from naive CD25-CD4+ T cells and (D) expression of CD45 isoforms upon 6 days of culture with plate-bound anti-CD3 antibodies (plate coated at 1 μg mL^−1^), plate-bound anti-CD3 antibodies/soluble anti-CD28 antibodies (0.1 μg mL^−1^), recombinant human (rh) IL-2 (100 U mL^−1^), or rhIL-7 (10 ng mL^−1^). Data are representative for experiments with three different donors. Statistical significance is indicated as ** *P* < 0.01, and *** *P* < 0.001 by Wilcoxon signed-rank test.

Although peripheral blood CD45RA+CD25^dim^CD4+ T cells lacked expression of CD69, we found evidence for prior *in vivo* activation of these cells. Already in our first analysis (Fig.2A), a somewhat lower per-cell expression level of CD45RA was noted on CD45RA+CD25^dim^CD4+ T cells than on naive CD25-CD4+ T cells. CD45RA to CD45RO transgression typically occurs upon TCR stimulation of naive T cells (Kristensson *et al*., 1992; Geginat *et al*., 2001). Analysis of CD45 isoforms confirmed the reduced per-cell expression of CD45RA, and slightly enhanced expression of CD45RO, on CD45RA+CD25^dim^CD4+ T cells when compared to naive CD25-CD4+ T cells (Fig.3A and data not shown). Although the CD45RO expression on CD45RA+CD25^dim^CD4+ T cells was markedly lower than on memory CD4+ T cells, we also observed that around 20% of CD45RA+CD25^dim^CD4+ T cells demonstrated a CD45RA^int^CD45RO^int^ phenotype vs. only 10% of naive CD25-CD4+ T cells (Fig.3B). These data imply prior *in vivo* TCR engagement of CD45RA+CD25^dim^CD4+ T cells.

Next, we sought to obtain *in vitro* evidence that TCR-derived signals drive the development of CD45RA+CD25^dim^CD4+ T cells. Indeed, CD45RA+CD25^dim^ cells developed from naive CD25-CD4+ T cells upon stimulation by anti-CD3 antibodies only (Fig.3C). These *in vitro* CD45RA+CD25^dim^CD4+ T cells also demonstrated slightly modulated expression of CD45 isoforms (Fig.3D). In contrast, combined CD3/CD28 cross-linking largely resulted in complete differentiation of naive CD25-CD4+ T cells into CD45RA-CD45RO+ memory cells and high CD25 expression (Fig.3C and D). Neither IL-2 (Fig.3C) nor IL-15 (data not shown) induced CD25 expression on CD25- naive CD4+ T cells. IL-7 readily induced CD25 expression on naive CD25-CD4+ T cells (Fig.3C), as previously reported (Cimbro *et al*., 2012; Pekalski *et al*., 2013). However, the per-cell CD25 expression of IL-7 stimulated cells was higher than typically observed for ex vivo analyzed CD45RA+CD25^dim^CD4+ T cells. Furthermore, IL-7 did not modulate CD45 isoform expression (Fig.3D). These combined data indicate that CD45RA+CD25^dim^CD4+ T cells develop from naive CD25-CD4+ T cells upon TCR stimulation only.

### CD45RA+CD25^dim^CD4+ T cells also reside in the CD31+ naive CD4+ T cell pool

Next, we assessed the relation between CD45RA+CD25^dim^CD4+ T cells and the previously reported population of CD31- central naive CD4+ T cells (Kohler *et al*., 2005). In accordance with the notion of prior TCR engagement, CD45RA+CD25^dim^CD4+ T cells demonstrated less CD31 expression than naive CD25-CD4+ T cells (Fig. S4A and B). Consequently, a substantial proportion of CD45RA+CD25^dim^CD4+ T cells were found in the CD31- central naive CD4+ T cell population (Fig. S4C). However, a significant proportion of CD45RA+CD25^dim^CD4+ T cells were also found among the CD31+ recent thymic emigrant naive CD4+ T cell population. The presence of these cells within the CD31+ naive CD4+ T cell fraction was not surprising, as we found that CD31 is only gradually lost upon CD3/CD28 cross-linking of CD31+ naive CD4+ T cells (Fig. S4D). Thus, the expression of CD25 reveals further heterogeneity among naive human CD4+ T cells.

### CD45RA+CD25^dim^CD4+ T cells develop in secondary lymphoid tissues

**Fig 4:**
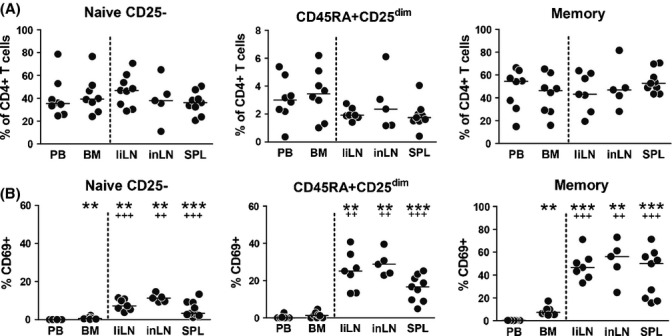
CD45RA+CD25^dim^CD4+ T cells develop in secondary lymphoid tissues. (A) Naive CD25-, CD45RA+CD25^dim^, and memory CD4+ T cells were enumerated by flow cytometry in mononuclear cell fractions isolated from peripheral blood (PB) and paired bone marrow (BM) samples, and from nonpaired liver-draining lymph node (liLN), inguinal lymph node (inLN), and spleen (SPL) samples.. (B) Expression of the activation marker CD69 by naive CD25-, CD45RA+CD25^dim^, and memory CD4+ T cells in peripheral blood and lymphoid tissues. Statistical significance was tested with Wilcoxon signed-rank test or Mann–Whitney U-test. Statistical significance vs. peripheral blood is indicated as **P < 0.01, ***P < 0.001. Statistical significance vs. bone marrow is indicated as ^++^ *P* < 0.01, ^+++^ *P* < 0.001.

As naive CD4+ T cells continuously circulate through lymphoid organs to encounter antigens, we hypothesized that CD45RA+CD25^dim^CD4+ T cells would develop in these organs. Analysis of mononuclear cells from human bone marrow, spleen, liver-draining lymph nodes, and inguinal lymph nodes obtained from young and intermediate age donors (median age 42, range 14–62) showed that naive CD25-CD4+ T cells, CD45RA+CD25^dim^CD4+ T cells, and memory CD4+ T cells were all present in these tissues (Fig.4A). No correlation between tissue CD45RA+CD25^dim^CD4+ T cells and age was observed, possibly due to the limited number of samples available. Next, we analyzed the expression of CD69, an activation marker that is induced on naive CD4+ T cells after TCR stimulation rather than cytokine stimulation (Simms & Ellis, 1996; Cimbro *et al*., 2012). Whereas T cells hardly expressed CD69 in peripheral blood and bone marrow, we observed prominent expression of CD69 on T cells in secondary lymphoid organs (Fig.4B). The percentage of CD69-expressing cells was highest among memory CD4+ T cells in lymph nodes and spleen, whereas only few naive CD25-CD4+ T cells expressed CD69 in these secondary lymphoid organs. Interestingly, a substantial part of CD45RA+CD25^dim^CD4+ T cells in lymph nodes and spleen showed expression of CD69. This suggests that TCR-derived signals drive the development of CD45RA+CD25^dim^CD4+ T cells in secondary lymphoid organs.

### Evidence for low-affinity TCR engagement of CD45RA+CD25^dim^CD4+ T cells

Current evidence from animal studies implies that low-affinity TCR engagement by peptide/MHC complexes promotes the homeostatic maintenance of naive T cells (Surh & Sprent, 2008). Therefore, we next hypothesized that CD45RA+CD25^dim^CD4+ T cells have undergone low-affinity TCR engagement *in vivo*. An accepted method to study the strength of TCR and peptide/MHC interactions is measurement of CD3 Ϛ chain phosphorylation levels (Mandl *et al*., 2013). As this procedure requires immediate analysis of T cell samples, it is incompatible with the currently employed T cell sorting strategy. Therefore, we decided to analyze expression levels of the inhibitory receptor CD5, which reflects prior TCR signaling strength (Mandl *et al*., 2013). In line with the notion of low-affinity TCR stimulation, CD45RA+CD25^dim^CD4+ T cells expressed low levels of CD5 (median MFI 54, range 48-98) which were substantially lower than in memory CD4+ T cells (median MFI 67, range 55-121) and comparable to that of naive CD25-CD4+ T cells (median MFI 54, range 12-101), as shown in Fig. S5. The combined data suggest that CD45RA+CD25^dim^CD4+ T cells develop upon low-affinity TCR–peptide/MHC interaction *in vivo*.

### CD45RA+CD25^dim^CD4+ T cells display increased sensitivity to IL-2

**Fig 5:**
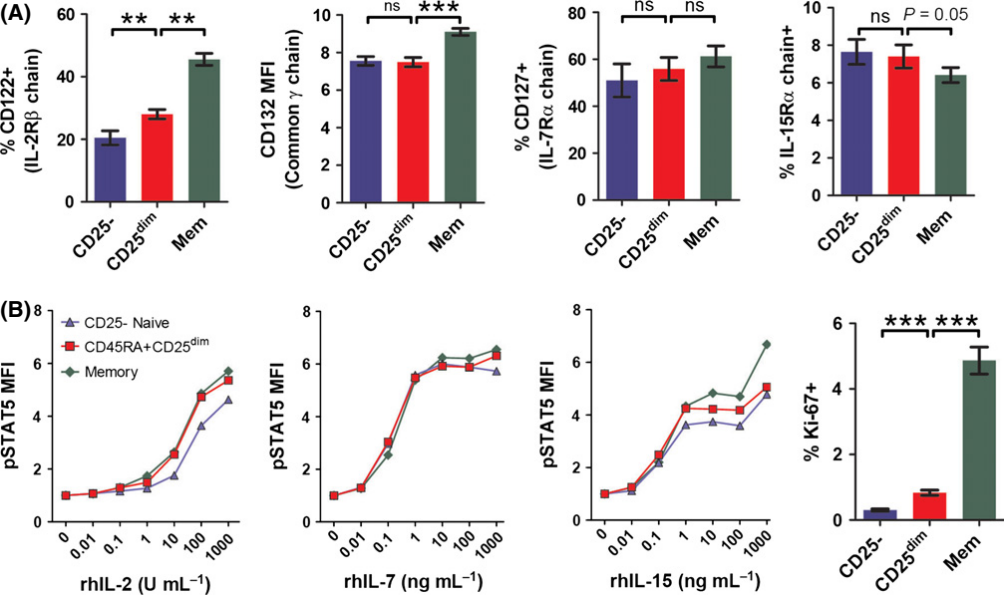
Increased sensitivity for IL-2 in CD45RA+CD25^dim^ CD4+ T cells. (A) Percentages of cells expressing CD122 (IL-2Rβ chain, *n *= 9), CD127 (*n *= 7), and the IL-15Rα chain (*n *= 9), and the mean fluorescence intensity (MFI) for CD132 (*n *= 13) in naive CD25-, CD45RA+CD25^dim^, and memory CD4+ T cells. For CD132, the MFI is shown, as all CD4+ T cells expressed CD132. (B) MFI for pSTAT5 in naive CD25-, CD45RA+CD25^dim^, and memory CD4+ T cells in response to increasing concentrations of recombinant human IL-2 (rhIL-2), IL-7 (rhIL-7), and IL-15 (rhIL-15). Mean values from experiments with cells from three donors are shown. Percentage of Ki-67-expressing cells (right panel) among naive CD25-, CD45RA+CD25^dim^ and memory CD4+ T cells of 29 healthy aged individuals. Bars and whiskers represent mean with SEM. Statistical significance is indicated as **P < 0.01, ***P < 0.001, by Wilcoxon signed-rank test. ns = nonsignificant.

Common γ-chain cytokines such as IL-2, IL-7, and IL-15 may support the maintenance of T cells by promoting STAT5-dependent proliferation and survival (Rochman *et al*., 2009). In addition to the IL-2Rα chain (CD25), CD45RA+CD25^dim^CD4+ T cells also expressed slightly more IL-2/IL-15Rβ chain (CD122) than naive CD25-CD4+ T cells (Figs5A and S6A). In contrast, expression of the common γ-chain (CD132), IL-7Rα chain (CD127), and IL-15Rα chain was similar in CD45RA+CD25^dim^CD4+ T cells and naive CD25-CD4+ T cells. Based on these findings, we predicted that CD45RA+CD25^dim^CD4+ T cells would be more responsive to IL-2 and perhaps IL-15 when compared to naive CD25-CD4+ T cells. CD45RA+CD25^dim^CD4+ T cells indeed were more sensitive to IL-2 than naive CD25-CD4+ T cells, as shown by more IL-2 induced STAT5 phosphorylation. (Figs5B and S6B). CD45RA+CD25^dim^CD4+ T cells were also slightly more sensitive to IL-15 than naive CD25-CD4+ T cells. In contrast, IL-7 induced similar STAT5 phosphorylation in CD45RA+CD25^dim^CD4+ T cells and naive CD25-CD4+ T cells. As additional sensitivity to IL-2 and IL-15 may provide extra signals to undergo proliferation (Rochman *et al*., 2009), we next compared the expression of the proliferation marker Ki-67 in CD45RA+CD25^dim^CD4+ T cells and naive CD25-CD4+ T cells. As expected, the percentage of proliferating cells was slightly higher among directly analyzed *ex vivo* CD45RA+CD25^dim^CD4+ T cells than naive CD25-CD4+ T cells (Fig.5B).

### CD45RA+CD25^dim^CD4+ T cells represent a broad and functional reservoir of naive T cells

**Fig 6:**
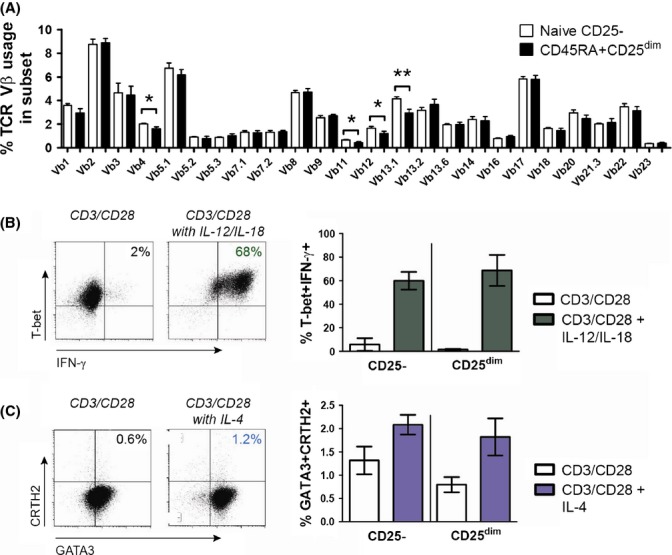
CD45RA+CD25^dim^ CD4+ T cells constitute a broad and functional reservoir of naive T cells. (A) Analysis of TCR Vβ usage in naive CD25- and CD45RA+CD25 ^dim^ CD4+ T cells of 9 aged individuals. (B) Representative T-bet and IFN-γ staining in CD45RA+CD25^dim^ CD4+ T cells (left panel) and percentages of T-bet+IFN-γ+ Th1 cells among naive CD25- and CD45RA+CD25^dim^ CD4+ T cells of 3 aged individuals (right panel) after 6 days of culture with anti-CD3/CD28-coated beads with/without IL-12+IL-18 (each 10 ng mL^−1^) and neutralizing anti-IL-4 antibodies. Cells were restimulated with PMA and Ca2+ ionophore in the presence of brefeldin A for 4 h on the final day. (C) Representative GATA-3 and CRTH2 staining in CD45RA+CD25^dim^ CD4+ T cells (left panel) and percentages of GATA-3+CRTH2+ Th2 cells among naive CD25- and CD45RA+CD25^dim^ CD4+ T cells of 3 aged individuals (right panel) after 6 day culture with anti-CD3/CD28-coated beads with/without IL-4 (25 ng mL^−1^) and anti-IFN-γ antibodies. Statistical significance is indicated as * *p* < 0.05 and ** *p* < 0.01, by Wilcoxon signed rank test.

As a broad TCR repertoire is essential for optimal immunity, we next studied the TCR repertoire of CD45RA+CD25^dim^CD4+ T cells. Although CD45RA+CD25^dim^CD4+ T cells showed subtle differences in TCR Vβ usage when compared to naive CD25-CD4+ T cells, CD45RA+CD25^dim^CD4+ T cells still demonstrated a broad TCR Vβ repertoire (Fig.6A).

Subsequently, we tested the ability of CD45RA+CD25^dim^CD4+ T cells to differentiate into memory T cells. CD45RA+CD25^dim^CD4+ T cells readily differentiated into CD45RO+ memory cells upon *in vitro* CD3/CD28 stimulation (Fig. S7). As CD45RA+CD25^dim^CD4+ T cells were not blocked in their development, we assessed whether CD45RA+CD25^dim^CD4+ T cells were capable of acquiring T helper (Th) cell effector functions. When cultured under Th1-polarizing conditions, CD45RA+CD25^dim^CD4+ T cells differentiated into IFN-γ+T-bet+ T helper 1 (Th1) cells (Fig.6B). The Th1-polarizing potential of CD45RA+CD25^dim^CD4+ T cells was similar to that of naive CD25-CD4+ T cells. CD45RA+CD25^dim^CD4+ T cells and naive CD25-CD4+ T cells also showed a similar ability to differentiate into GATA3+CRTH2+ T helper 2 (Th2) cells (De Fanis *et al*., 2007) under Th2-polarizing conditions (Fig.6C). In aggregate, our data show that CD45RA+CD25^dim^CD4+ T cells constitute a broad and functional reservoir of naive-like CD4+ T cells in aged humans.

## Discussion

Here, we report on a unique mechanism of post-thymic maintenance of human naive CD4 T cells, which was not observed for naive CD8 T cells. We found that a robust population of naive CD4 T cells with evidence of prior *in vivo* TCR engagement develops as a function of age in healthy individuals. We show that this subset, defined by increased CD25 expression, likely develops in secondary lymphoid organs as a result of low-affinity TCR engagement and is further maintained by IL-2. Thus, as thymic output wanes, IL-2 becomes an important homeostatic cytokine for the peripheral maintenance of naive CD4+ T cells in aged humans. We conclude that these CD25 expressing cells represent an important reservoir of naive-like cells that contributes to immunity in the elderly.

Recently, increased proportions of CD25-expressing naive CD4+ T cells were observed in aged individuals (Pekalski *et al*., 2013). We here extend these findings using a state-of-the-art flowcytometry strategy (Miyara *et al*., 2009, 2011) and identified these cells as CD45RA+CD25^dim^CD4+ T cells in the peripheral blood of aged humans. A major advantage of our strategy is the possibility to delineate the aging-associated CD45RA+CD25^dim^CD4+ T cells from conventional naive CD25-CD4+ T cells and naive CD25^int^ Treg cells. We precluded that CD45RA+CD25^dim^CD4+ T cells are late-stage memory CD4+ T cells that re-express CD45RA (Akbar & Henson, 2011; Di Mitri *et al*., 2011) and also confirmed their naive phenotype by analyzing differentiation markers, homing receptors, and intracellular cytokine production. Although the proportional increase in CD45RA+CD25^dim^CD4+ T cells could merely result from the decrease in the other naive CD4+ T cell fractions, we also found that the absolute number of CD45RA+CD25^dim^CD4+ T cells increased with age. Our study is therefore the first to show that aging is associated with a genuine increase in CD25-expressing naive CD4+ T cells.

Recently, the accumulation of CD25-expressing naive CD4+ T cells was explained by IL-7-mediated expansion of these cells (Pekalski *et al*., 2013). We now provide evidence that CD45RA+CD25^dim^CD4+ T cells primarily develop from naive CD25-CD4+ T cells upon low-affinity TCR engagement. CD45RA+CD25^dim^CD4+ T cells showed a slight shift in CD45 isoform expression (i.e., from CD5RA to CD45RO) and less CD31 expression in comparison with naive CD25-CD4+ T cells. Changes in expression of CD45 isoforms and CD31 on naive CD4+ T cells occur after TCR engagement rather than IL-7 stimulation (Kohler *et al*., 2005; Cimbro *et al*., 2012). Furthermore, typical CD45RA+CD25^dim^CD4+ T cells developed *in vitro* from naive CD25-CD4+ T cells after stimulation with anti-CD3 antibodies only. Although we confirmed that IL-7 can induce CD25 expression on naive CD25-CD4+ T cells, the per-cell expression of CD25 was much higher than typically observed on directly ex vivo analyzed CD45RA+CD25^dim^CD4+ T cells. Furthermore, IL-7 did not modulate CD45 isoform expression. An important role for IL-7 in the development of CD45RA+CD25^dim^CD4+ T cells in aged humans also seems unlikely, as IL-7 levels decline with age (Kang *et al*., 2004; Banerjee *et al*., 2011). Taken together, our data imply that TCR-derived signals rather than IL-7 drive the development of CD45RA+CD25^dim^CD4+ T cells in humans.

CD45RA+CD25^dim^CD4+ T cells likely receive TCR-derived signals in secondary lymphoid tissues. CD45RA+CD25^dim^CD4+ T cells showed substantial expression of the activation marker CD69 in human lymph nodes and spleen, but not in the peripheral blood and bone marrow. Importantly, CD69 is induced on naive T cells after TCR stimulation, but not cytokine stimulation (Simms & Ellis, 1996; Cimbro *et al*., 2012). Recently, regulatory T cells and conventional memory T cells were reported to express CD69 in secondary lymphoid tissues (Peters *et al*., 2013; Sathaliyawala *et al*., 2013). Although commonly used as an activation marker, CD69 may actually play a role in retention of activated T cells in lymphoid tissues via downmodulation of the sphingosine-1-phosphate receptor-1 (Shiow *et al*., 2006). This could explain the absence of CD69-expressing CD45RA+CD25^dim^CD4+ T cells in the circulation.

The exact nature of the peptide/MHC complexes involved in the development of human CD45RA+CD25^dim^CD4+ T cells remains unclear. Animal studies indicate that endogenous peptides may be involved in this process. Depriving mouse naive T cells of self-peptide/MHC complexes typically leads to naive T cell apoptosis (Sprent & Surh, 2011). Non-self (i.e., microbial)-peptides are unlikely to play an important role in the maintenance of the naive CD4+ T cell repertoire, as such peptides are usually offered to T cells in the presence of costimulatory signals. TCR triggering in the presence of costimulation typically leads to full memory T cell differentiation, as also demonstrated in the current study. Although we confirmed that CMV may have a profound impact on the maintenance of the naive CD4+ T cell pool (Wertheimer *et al*., 2014), we here precluded CMV as a driving force behind the accumulation of CD45RA+CD25^dim^CD4+ T cells.

Our study reveals further heterogeneity within the human naive CD4+ T cell pool. Previously, the naive CD4+ T cell compartment was mainly classified into nonprimed CD31+ thymic naive cells and primed CD31- central naive cells (Kimmig *et al*., 2002). Although CD45RA+CD25^dim^CD4+ T cells were abundant in the CD31- naive population, we also observed CD45RA+CD25^dim^CD4+ T cells in the CD31+ naive population. The presence of CD45RA+CD25^dim^CD4+ T cells within the supposedly non-TCR-engaged CD31+ naive population was not surprising, as our data demonstrated that CD31 is only gradually lost upon CD3/CD28 stimulation. The latter finding is in accordance with Demeure *et al*., showing that T cells loose CD31 only after multiple rounds of CD3/IL-2 stimulation (Demeure *et al*., 1996). Thus, in addition to the surface marker CD31 (Kohler & Thiel, 2009), the expression of CD25 allows for the identification of naive-like CD4+ T cells with a history of prior TCR engagement.

Whereas primarily IL-7 is considered important for the homeostasis of naive T cells (Rochman *et al*., 2009), CD45RA+CD25^dim^CD4+ T cells showed enhanced sensitivity to IL-2, and to some extent IL-15. IL-2 and IL-15 are also involved in the homeostasis of memory T cells and regulatory T cells (Rochman *et al*., 2009). Cytokines such as IL-2 and IL-15 can induce STAT5-dependent proliferation in T cells (Rochman *et al*., 2009). Interestingly, circulating CD45RA+CD25^dim^CD4+ T cells demonstrated a slightly higher proliferation rate than naive CD25-CD4+ T cells. The latter finding may be relevant as naive CD4+ T cells are long-lived cells that may undergo slow but substantial homeostatic proliferation over a longer period of time (den Braber *et al*., 2012). As serum levels of IL-2 and IL-15 are retained in the elderly (Kang *et al*., 2004; Gangemi *et al*., 2005; Banerjee *et al*., 2011; Kim *et al*., 2011), the enhanced sensitivity for IL-2 (and IL-15) may be an important adaptation of naive CD4+ T cells to the changing cytokine milieu in aged humans (Fig. S8).

CD45RA+CD25^dim^ cells did not accumulate within the naive CD8+ T-cell compartment of aged humans. The absence of naive CD45RA+CD25^dim^CD8+ T cells could suggest that IL-2 has no role in the peripheral maintenance of naive CD8+ T cells. However, several reports have shown that IL-2 imposes strong stimulatory effects on naive CD8+ T cells (Cho *et al*., 2007, 2013; Kamimura & Bevan, 2007). IL-2 not only promotes the proliferation of naive CD8+ T cells, but can also drive the differentiation into memory cells (Cho *et al*., 2007; Kamimura & Bevan, 2007). Therefore, the absence of naive CD45RA+CD25^dim^ CD8+ T cells and the pronounced decline in naive CD8+ T cells in aged humans could be related to the relatively low threshold of naive CD8+ T cells to convert into memory cells. Indeed, aging is associated with a substantial shift from naive CD8+ T cells toward central and effector memory cells (Wertheimer *et al*., 2014).

Our data imply that CD45RA+CD25^dim^CD4+ T cells may contribute to immunity in aged humans. CD45RA+CD25^dim^CD4+ T cells represent a broad and functional reservoir of naive CD4+ T cells in aged humans. CD45RA+CD25^dim^CD4+ T cells showed broad TCR Vβ usage and a similar ability to differentiate into Th1 and Th2 cells as naive CD25-CD4+ T cells. Importantly, our *in vitro* experiments show that CD45RA+CD25^dim^CD4+ T cells can be generated from naive CD25-CD4+ T cells. Furthermore, prior studies with IL-2 therapy have shown that nonregulatory CD25-expressing naive CD4+ T cells can be expanded by intermittent IL-2 therapy (Natarajan *et al*., 2002; Sereti *et al*., 2002, 2004). Enhancing circulating numbers of CD45RA+CD25^dim^CD4+ T cells could therefore be an interesting strategy for preserving or restoring immunity in aged humans.

In conclusion, our study shows that TCR engagement drives the emergence and accumulation of CD25-expressing naive CD4+ T cells in healthy aged humans. These cells, which likely develop in secondary lymphoid tissues, represent a broad and functional reservoir of naive CD4+ T cells in aged humans. Our study provides new insight into the homeostasis of human, naive CD4+ T cells and justifies further studies into CD4+ T cell expanding treatments to promote immunity in aged and immune compromised humans.

## Experimental procedures

### Donor samples and study approval

In a cross-sectional study, blood samples were obtained from 91 healthy individuals (age 20–92). Health was assessed by a health assessment questionnaire, a physical examination, and blood tests. Only a slight elevation in blood pressure and the use of antihypertensive treatment were accepted. Also, clinical and laboratory data attesting to the donors’ overall health were assessed. In addition, blood/bone marrow samples were obtained from 8 healthy stem cell donors, before mobilization with G-CSF, spleen samples from 9 deceased kidney donors, liver-draining lymph nodes from 7 deceased liver donors, and inguinal lymph nodes from 5 kidney transplant recipients at the time of transplantation (not treated with immunosuppressive drugs prior to lymph node excision). Single cell suspensions were obtained from tissues as previously described (Peters *et al*., 2013). Mononuclear cell fractions were isolated by density gradient centrifugation with Lymphoprep (Axis-Shield and Nycomed Pharma). Written informed consent was obtained from all study participants or their representatives, and the study was approved by medical ethical committees at the participating study centers.

### Flow cytometry

Isolated mononuclear cells or whole blood samples were stained with the following fluorochrome-conjugated monoclonal antibodies: CD4-PcP, CD8-APC-H7, CD31-PE, CD45RA-FITC, CD25-PE, CD45RO-PE-Cy7, CD45RO-FITC, CCR7-PcP-Cy5.5, TCRγδ-BV421, pSTAT5-PE-Cy7, Ki-67-PcP-Cy5.5, Tbet-PE, CXCR3-PE-Cy5, CD69-APC-Cy7, CCR4-PE-Cy7, CD5-APC, CRTH2-PE, GATA3-APC, CD5-APC (all BD), CD4-ECD, CD4-PC7, CD69-PC5, CD69-ECD, Beta Mark TCR V β kit (all Beckman Coulter, Woerden, The Netherlands), CD122-PE, CD132-PE, CCR6-PcP-Cy5.5, IL-2-AF700, IL-4-PE, IFN-γ-PcP-Cy5.5, FOXP3-AF647, Helios-AF488 (all Biolegend, Uithoorn, The Netherlands), CD4-ef450, CD25-APC, CD25-PE, CD45RA-PE, CD45RA-ef605, HLA-DR-APC-ef780, IL-17-AF488, CD27-AF700, CD28-PcP-cy5.5 (all eBioscience, Vienna, Austria). In case of whole blood staining, samples were lysed with BD FACS lysing solution. Samples were measured on a LSR-II (BD) or FC500 (Beckman Coulter) and analyzed with Kaluza Analysis Software (Beckman Coulter). Absolute numbers of CD4+ and CD8+ T cells were determined according to the BD MultiTest TruCount method, as described by the manufacturer. TruCount measurements were taken on a FACS Canto-II (BD) and analyzed with FACSCanto Clinical Software (BD).

### Intracellular cytokine and transcription factor staining

Whole blood samples were diluted 1:1 with RPMI and stimulated with PMA and Ca2+ ionophore A23187 in the presence of brefeldin A (Sigma-Aldrich, Zwijndrecht, The Netherlands) for 4 h. After red blood cell lysis with ammonium chloride, cells were fixed and permeabilized with a Foxp3 Staining Buffer Set (eBioscience) followed by intracellular staining.

### Phosphorylated STAT5 staining

Peripheral blood mononuclear cells (PBMCs) were stimulated with indicated concentrations of recombinant human (rh) IL-2, rhIL-7, or rhIL-15 (all Peprotech) during 15 min, directly followed by fixation with Cytofix Buffer (BD) for 10 min at 37 °C. Subsequently, the cells were treated with Perm Buffer III (BD) for 30 min on ice and stained for pSTAT5.

### Cell culture

Naive T cells fractions of interest were sorted on a MoFlo Astrios sorter (Beckman Coulter). For assessment of CD31 loss, sorted CD31+ naive CD4+ T cells were stained with 5 μm proliferation dye CFSE. The cells were cultured for 6 days in 96-well flat-bottomed plates in RPMI1640 with 10% human pooled serum (HPS) and gentamycin. Cells were stimulated with CD3/CD28-coated beads (Life Technologies, Paisley, UK) at a cell to bead ratio of 1:1. For induction of CD45RA+CD25^dim^CD4+ T cells, sorted naive CD45RO-CCR7+CD25- CD4+ T cells were cultured for 6 days in the presence of plate-bound anti-CD3 antibodies (wells coated at concentration of 1 μg mL^−1^) with/without soluble anti-CD28 antibodies (0.1 μg mL^−1^), or with indicated concentrations of rhIL-2 or rhIL-7. For polarizing experiments, sorted CD45RA+CD25- and CD45RA+CD25^dim^ CD4+ T cells were cultured with CD3/CD28-coated beads at a cell to bead ratio of 1:1 in the presence or absence of rhIL-12 (10 ng mL^−1^), rhIL-18 (10 ng mL^−1^), and anti-IL-4 mAb (10 μg mL^−1^) for Th1 skewing; or rhIL-4 (25 ng mL^−1^) and anti-IFN-γ mAb (10 ng mL^−1^) for Th2 skewing. The recombinant human cytokines were purchased from Peprotech and blocking antibodies from BD. After 4 days, the medium was refreshed and all cytokines and blocking antibodies were added again. After 6 days, the cells were restimulated for 4 h with PMA and calcium ionophore in the presence of brefeldin A.

### CMV-specific antibodies

Serum levels of CMV-specific IgG were determined with an in-house ELISA. 96-well ELISA plates (Greiner) were coated with lysates of CMV-infected fibroblasts overnight. Lysates of noninfected fibroblasts were used as negative controls. Following the coating, dilutions of serum samples were incubated for 1 h. Goat anti-human IgG was added and incubated for 1 h. Samples were incubated with phosphatase for 15 min, and the reaction was stopped with NaOH. The plates were scanned on a Versamax reader (Molecular Devices). A pool of sera from 3 CMV-seropositive individuals with known concentrations of CMV-specific IgG was used to quantify CMV IgG in the tested samples.

## Supporting Information

**Fig. S1** Maintenance of naive CD4+ T cells and loss of naive CD8+ T cells in aged humans. **Fig. S2** Accumulation of CD45RA+CD25^dim^CD4+ T cells in peripheral blood of aged humans is not driven by sex or CMV serostatus. **Fig. S3** Circulating CD45RA+CD25^dim^CD4+ T cells demonstrate a naive-like phenotype. **Fig. S4** CD45RA+CD25^dim^CD4+ T cells show signs of prior TCR engagement. **Fig. S5** Per-cell expression of CD5 by CD45RA+CD25^dim^CD4+ T cells. **Fig. S6** Increased sensitivity for IL-2 in CD45RA+ CD25^dim^CD4+ T cells. **Fig. S7** Acquisition of CD45RO by anti-CD3/CD28 stimulated CD45RA+CD25^dim^CD4+ T cells. **Fig. S8** Schematic overview of naive CD4+ T cell maintenance in young and aged humans.
