# Supplementary material for: Low-affinity TCR engagement drives IL-2-dependent post-thymic maintenance of naive CD4+ T cells in aged humans
Source: Aging Cell. 2015 May 25;14(5):744–53. doi: 10.1111/acel.12353 (PMC4568962; doi:10.1111/acel.12353)

**Supportive Information ACE-14-0382 van der Geest et al.**

**Supplemental Figures 1-8 (see below)**

Supplemental Figure 1. Maintenance of naive CD4+ T cells and loss of naive CD8+ T cells in aged humans. Absolute numbers of (A) naive CD4+ and (B) naïve CD8+ T cells as a function of age in 52 CMV-seropositive (CMV+) and 39 CMV-seronegative (CMV-) healthy, adult humans. Donor ages ranged between 20 and 92. Correlations were tested with Spearman’s rank correlation coefficient and p values are shown in the legend of the graphs.


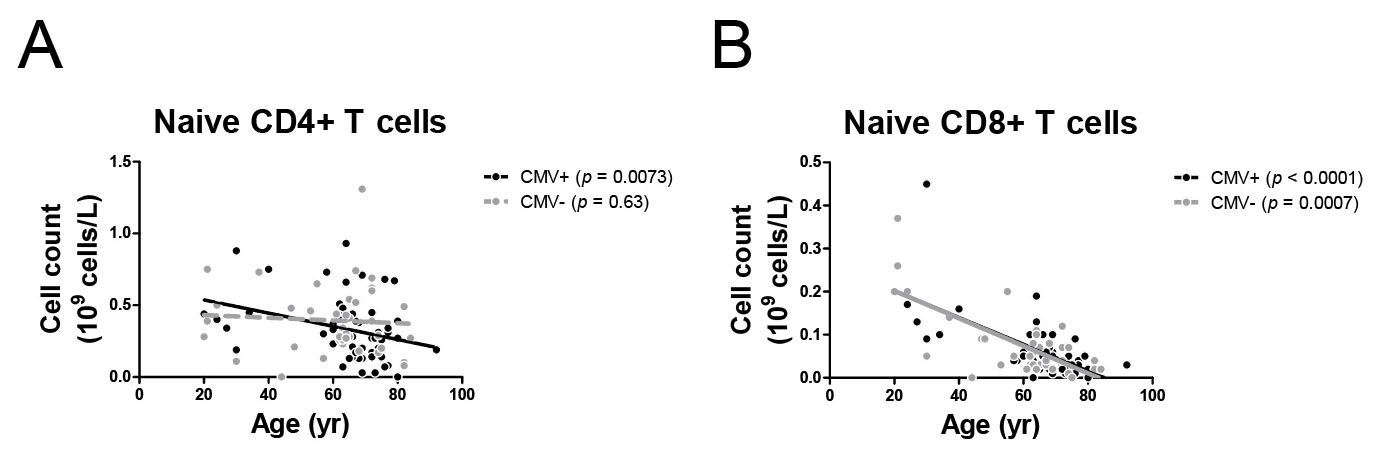


Supplemental Figure 2. Accumulation of CD45RA+CD25^dim^CD4+ T cells in peripheral blood of aged humans is not driven by sex or CMV serostatus. (A) Proportions of CD45RA+CD25^dim^CD4+ T cells in young (<40yr) and aged (>60yr) male and female individuals. **(B)** Proportions of CD45RA+CD25^dim^CD4+ T cells in CMV-seropositive (CMV+) aged individuals and age-matched, CMV-seronegative (CMV-) individuals. *** indicates *p*<0.001, by Mann Whitney U test.


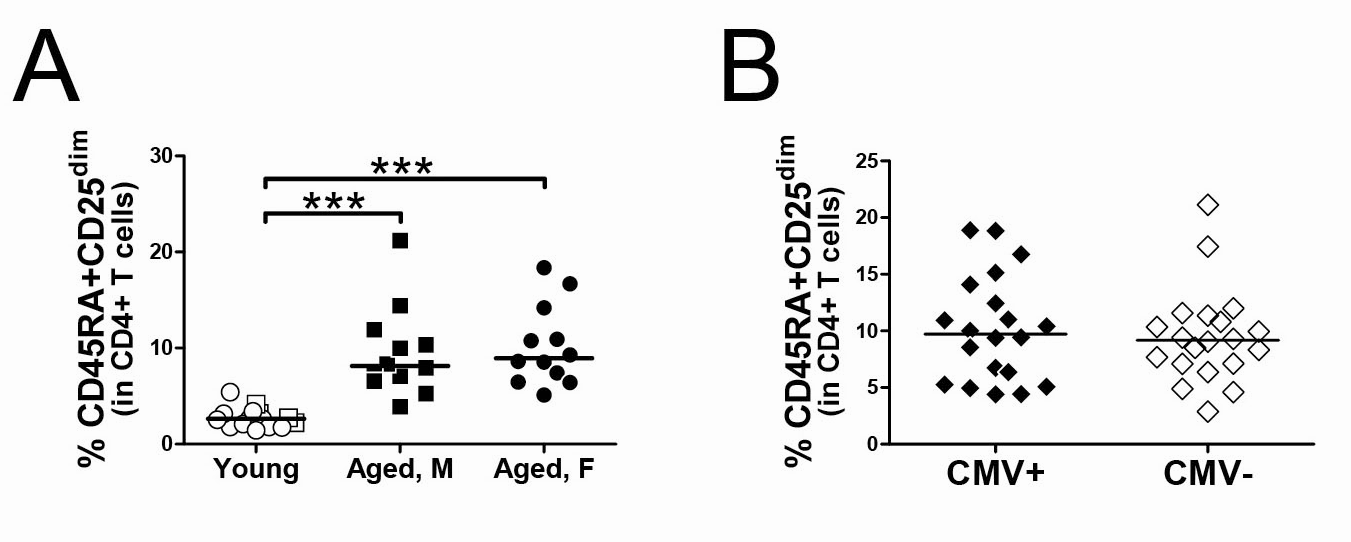


Supplemental Figure 3. Circulating CD45RA+CD25^dim^CD4+ T cells demonstrate a naive-like phenotype. Naive CD25-CD4+ T cells, CD45RA+CD25^dim^CD4+ T cells, naive CD25^int^ regulatory T cells and memory (Mem) CD4+ T cells or memory CD25^high^ regulatory T cells of aged individuals were assessed for expression of (A) CD27 and CD28, (B) homing receptors, (C) intracellular cytokines, (D) regulatory T cell markers and (E) the early activation marker CD69. Results are expressed as the percentage of cells expressing the specific markers. Intracellular cytokine production was studied after 4 hour stimulation with PMA and Ca2+ ionophore in the presence of Brefeldin A.


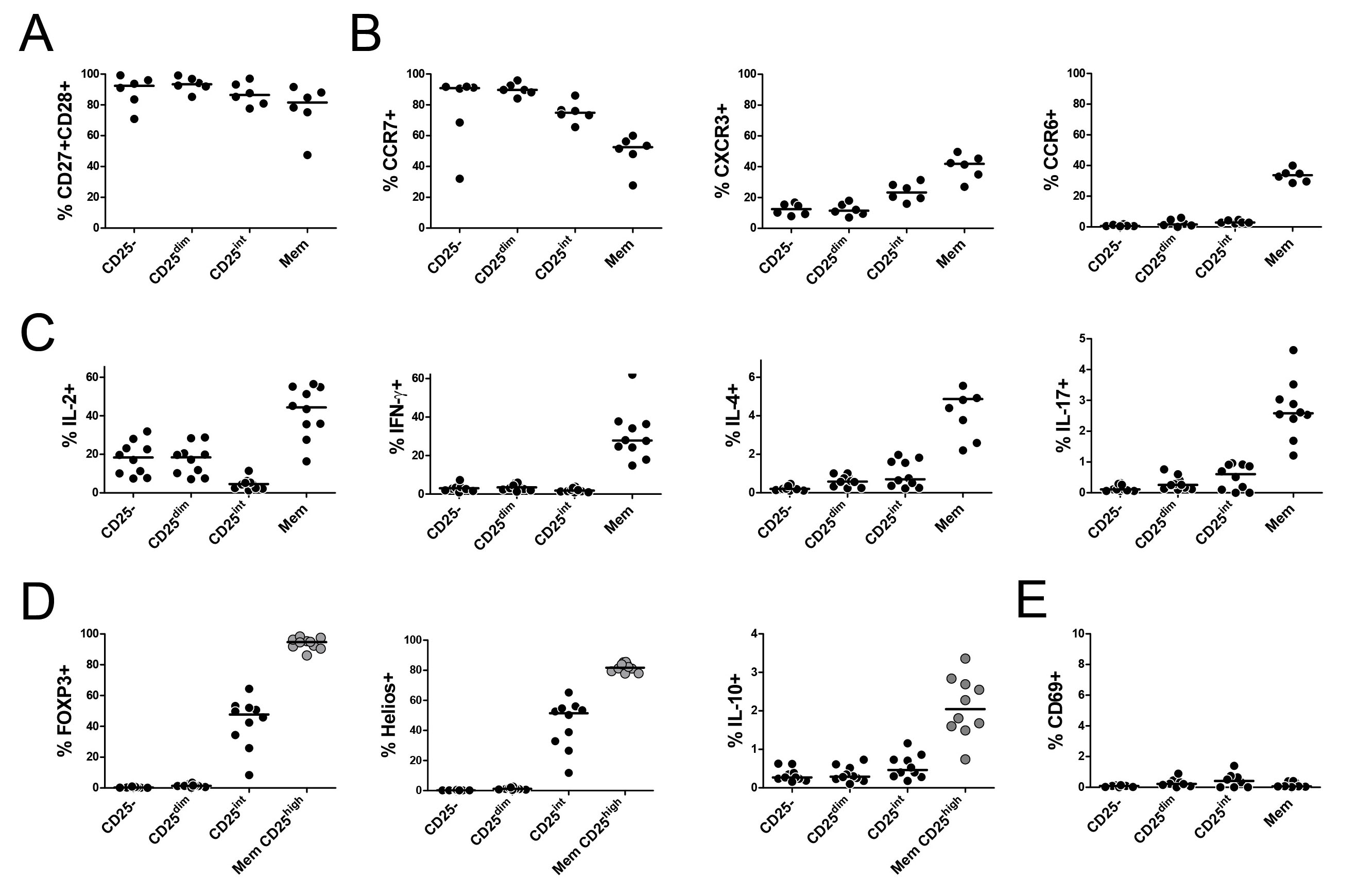


Supplemental Figure 4. CD45RA+CD25^dim^CD4+ T cells show evidence of prior TCR engagement. (A) Representative staining for CD31 in naive CD25-, CD45RA+CD25^dim^ and memory CD4+ T cells and (B) expression of CD31 in the 3 CD45RA+CD4+ T cell subsets and memory CD4+ T cells of 29 aged individuals. (C) Mean proportions of CD31+ and CD31- cells as well as CD25^dim^ and CD25- cells within the naive CD4+ T cell compartment of 11 young and 15 aged individuals. (D) CD31 expression on sorted, CFSE-labeled CD31+CD45RA+CCR7+ naive CD4+ T cell upon 6 days culture with anti-CD3/CD28 coated beads. Baseline (BL) cells were directly analyzed after sorting. Data represent experiments with 4 different donors. Bars and whiskers represent mean with SEM. Statistical significance is indicated as * p<0.05 and *** p<0.001, by Wilcoxon signed rank test.


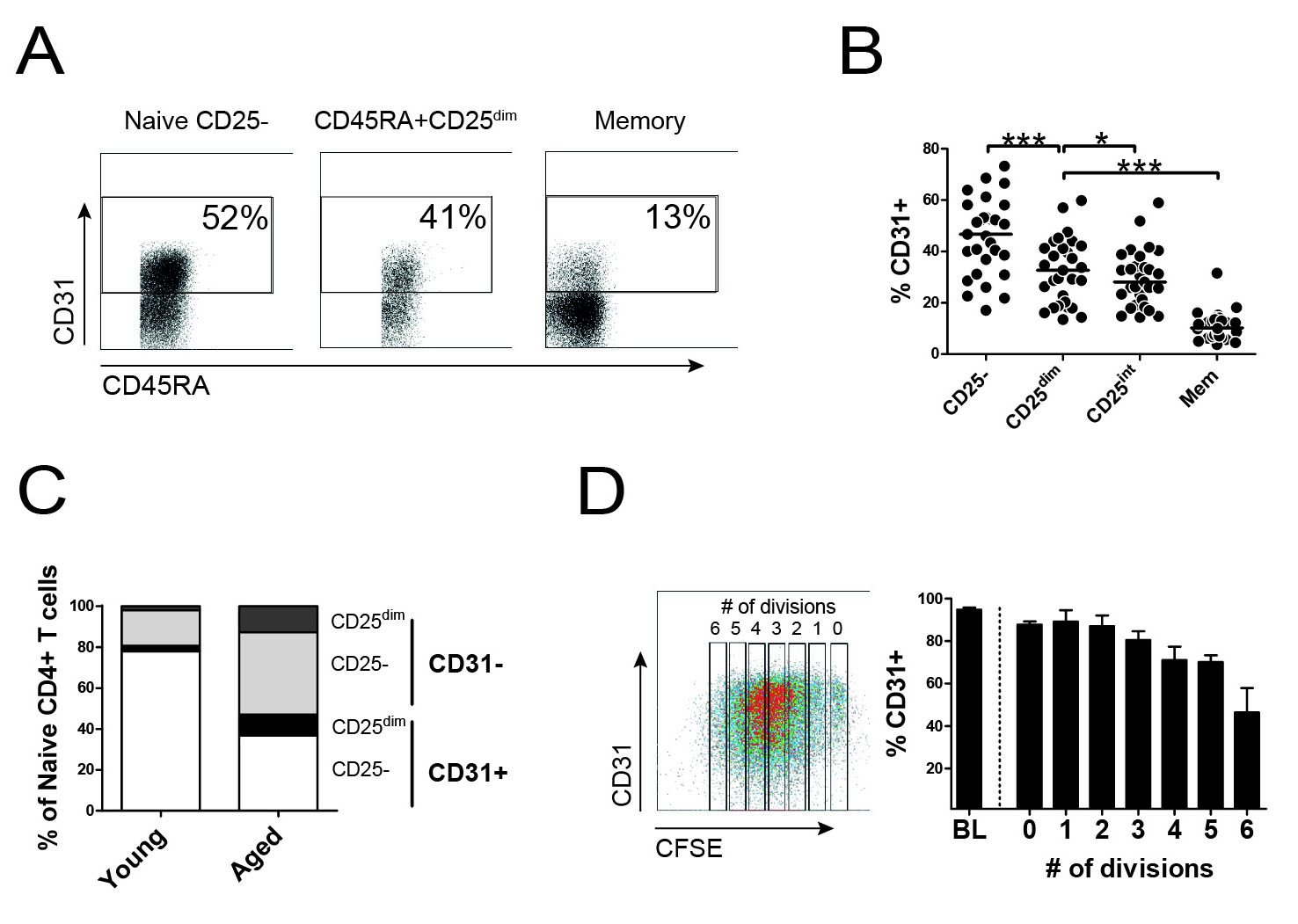


**Supplemental Figure 5. Per-cell expression of CD5 by CD45RA+CD25^dim^CD4+ T cells.** Mean fluorescence intensity (MFI) is shown for naive CD25-CD4+ T cells and CD45RA+CD25^dim^ CD4+ T cells, as well as memory CD4+ T cells of 8 aged individuals. Bars and whiskers represent median and interquartile range. Statistical significance is indicated as ** p<0.01 by Wilcoxon signed rank test. ns = non-significant.


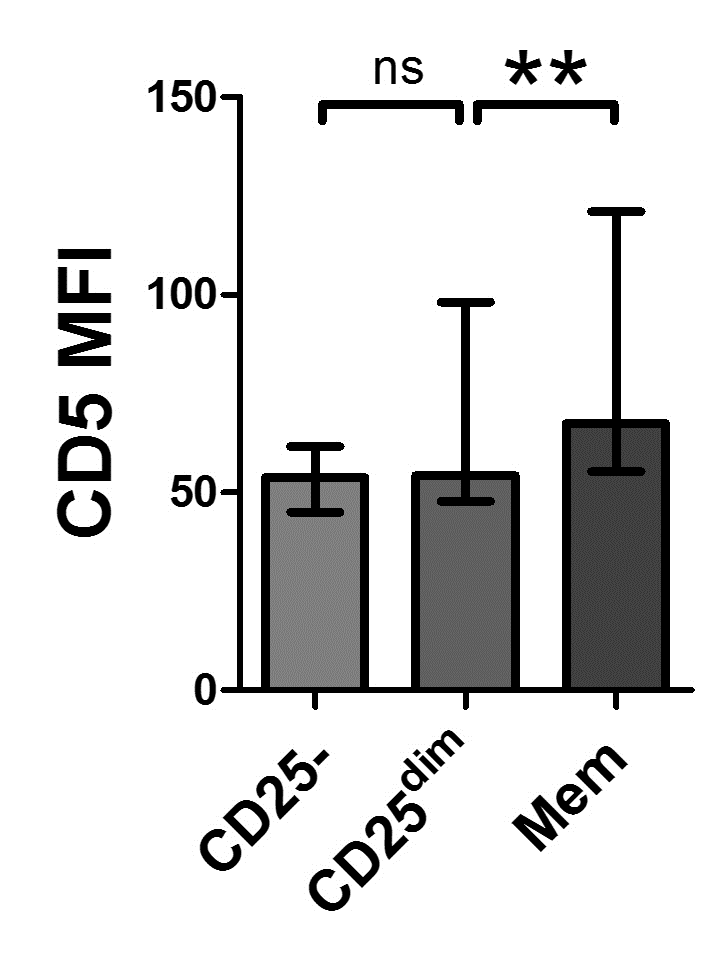


Supplemental Figure 6. Increased sensitivity for IL-2 in CD45RA+ CD25^dim^CD4+ T cells. (A) Representative flow cytometric staining for CD122 (IL-2Rβ chain), CD132 (common γ chain), CD127 (IL-7Rα chain) and the IL-15Rα chain in naive CD25-, CD45RA+CD25^dim^ and memory (mem) CD4+ T cells. (B) MFI for pSTAT5 in naive CD25-, CD45RA+CD25^dim^ and memory CD4+ T cells of aged individuals following 15 minutes stimulation with 100 U/mL IL-2 (n=9), 100 ng/mL IL-7 (n=8) or 100 ng/mL IL-15 (n=7). Bars and whiskers represent mean with SEM. Statistical significance is indicated as * p<0.05, ** and p<0.01 by Wilcoxon signed rank test. ns = non-significant.


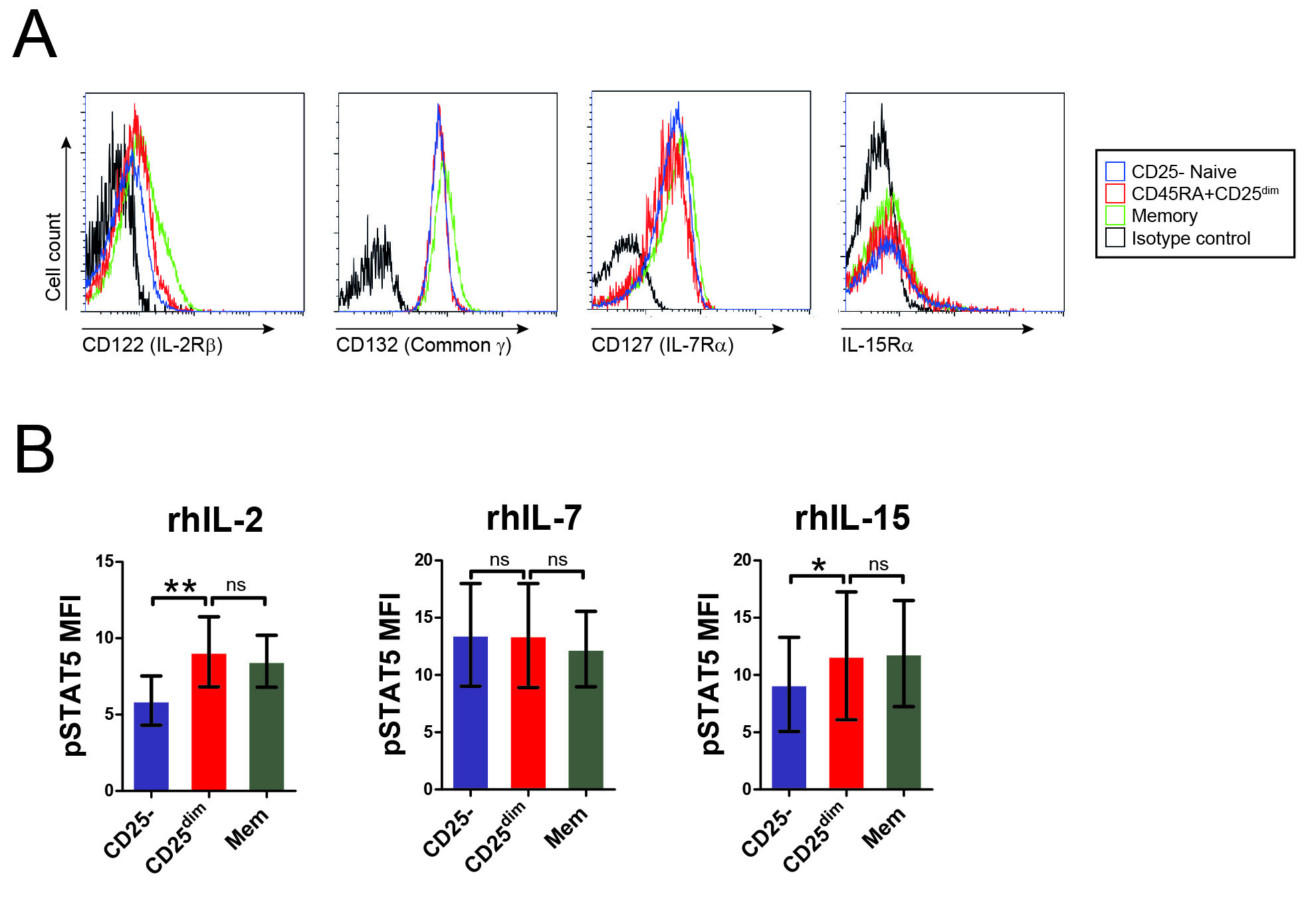


Supplemental Figure 7. Acquisition of CD45RO by anti-CD3/CD28 stimulated CD45RA+CD25^dim^CD4+ T cells. Differentiation of sorted naive CD25- and CD45RA+CD25^dim^CD4+ T cells into CD45RO+ memory cells upon 6 day culture with anti-CD3/CD28 coated beads. The sorted cells were obtained from 2 healthy, aged individuals.


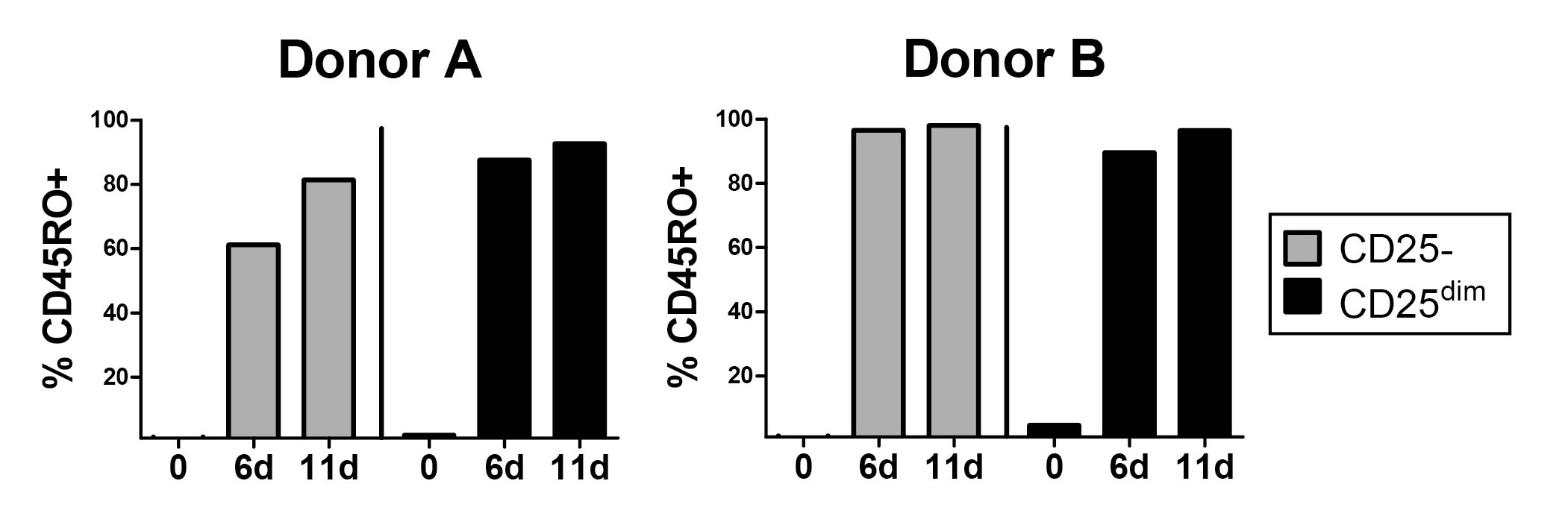


### Supplemental Figure 8. Schematic overview of naive CD4+ T cell maintenance in young and aged humans. Left panel: at young age, the naive CD4+ T cell pool is continuously replenished by new T cells from the thymus. These naive CD4+ T cells are maintained via IL-7. In addition, some naive CD4+ T cells receive TCR-derived signals in secondary lymphoid tissues, possibly via engagement with self-peptide/MHC complexes, and become CD45RA+CD25^dim^CD4+ T cells. These naive-like cells acquire enhanced sensitivity for IL-2. Right panel: at old age, thymic output has dropped due to thymic involution. What is left of the naive CD4+ T cell repertoire is partly maintained by IL-7. In addition, a substantial fraction of CD45RA+CD25^dim^CD4+ T cells accumulate over time, partly due to an enhanced sensitivity to IL-2. Of interest, serum levels of IL-2 are retained at old age, whereas levels of IL-7 decline.


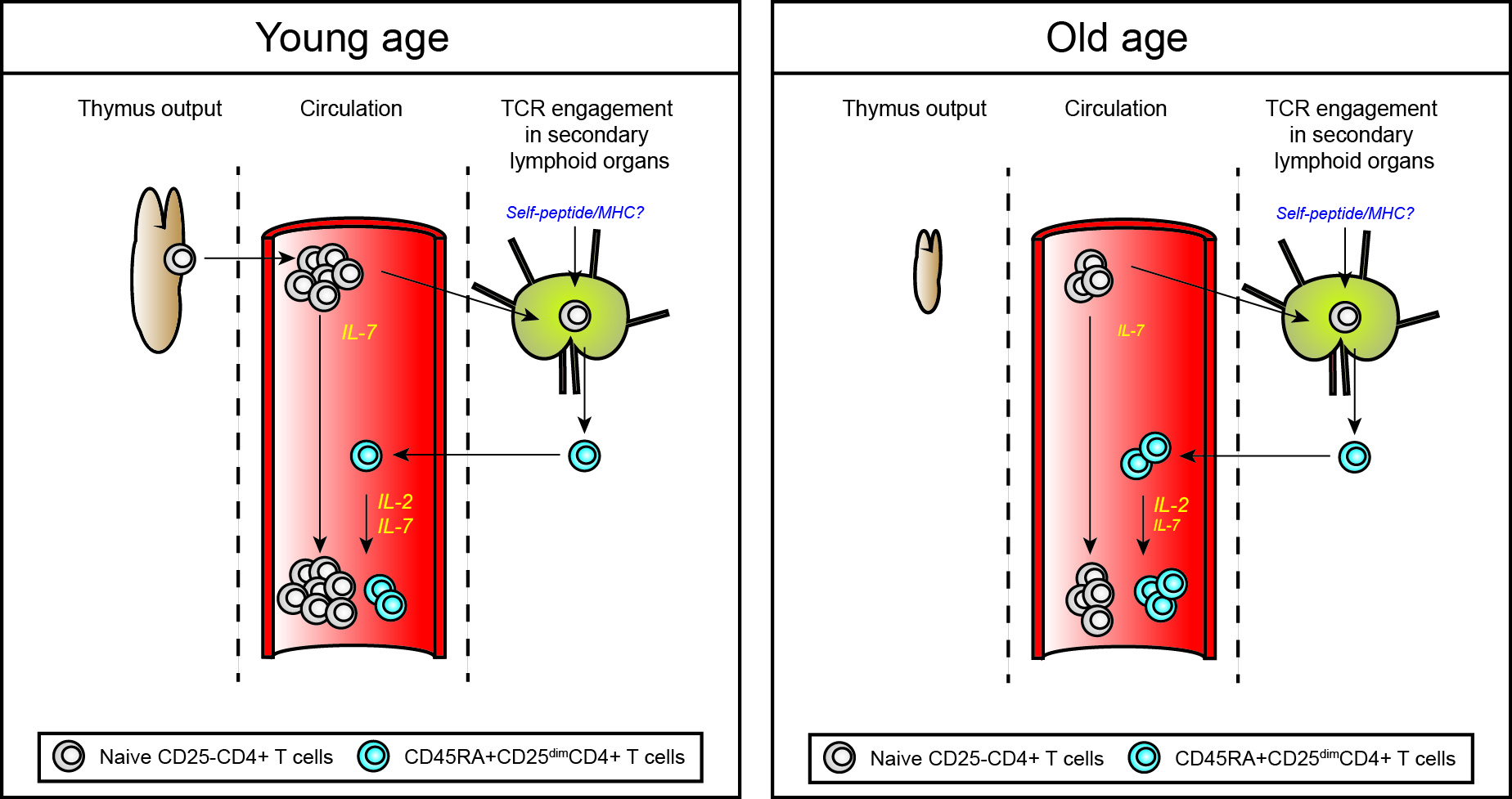

Supplement: Supplementary file 1 [file acel0014-0744-sd1.docx]
